# Nursing Care and Patient Education for Ulcerative Colitis Patients in Japan: A Scoping Review

**DOI:** 10.7759/cureus.102024

**Published:** 2026-01-21

**Authors:** Asuka Hashino, Kentaro Hara

**Affiliations:** 1 Department of Fundamental Nursing, Kumamoto University, Kumamoto, JPN; 2 Healthcare Management Research Center, Chiba University, Chiba, JPN; 3 Clinical Research Center, NHO (National Hospital Organization) Nagasaki Medical Center, Nagasaki, JPN

**Keywords:** nursing practice, patient education, scoping review, self-management, ulcerative colitis

## Abstract

Ulcerative colitis (UC) is a chronic inflammatory bowel disease marked by continuous inflammation of the mucosal lining of the rectum and colon, often leading to erosion and ulcer formation. The number of patients with UC in Japan has been increasing annually. The importance of self-care, which is crucial in the management of this chronic disease, has grown. This study aimed to assess the current status of nursing care and patient education provided by nurses to patients with UC in Japan through a scoping review.

We used the following search keywords: "ulcerative colitis", "nursing", "self-management", "patient education", and "diet therapy". We searched the Japan Medical Abstracts Society Web (Ichushi-Web) and CiNii databases. Based on predefined inclusion criteria, two researchers independently screened articles. From the selected studies, data on nursing care and patient education provided by nurses were extracted. Seven studies met the inclusion criteria, including six case reports and one survey study. The extracted content of nursing care and patient education was categorized into eight categories: Stoma Management and Care, Guidance on Self-Management of Medication and Wound Care, Collaboration and Coordination with Other Professionals, Pain Management, Wound and Ulcer Care, Assistance with Activities of Daily Living, Patient Understanding and Advocacy Role, and Psychological Care. These were further grouped into four overarching themes: Self-Management Education and Technical Guidance, Multidisciplinary Collaboration, Direct Care, and Patient Understanding and Emotional Support.

Nursing care and patient education for patients with UC may have clinical utility in improving quality of life and supporting continued treatment by alleviating patients' critical conditions, facilitating stoma acceptance, and providing appropriate wound care and pain management.

## Introduction and background

Ulcerative colitis (UC) is a chronic inflammatory disease characterized by continuous inflammation of the mucosal lining of the rectum and colon, often leading to erosion and ulcer formation. Similar to Crohn’s disease (CD), it is classified as an inflammatory bowel disease (IBD). Although genetic predisposition is considered a risk factor, environmental influences such as diet and infections are also believed to contribute to its onset; however, the precise etiology remains unclear.

Current treatment options for UC include pharmacological therapies, such as 5-aminosalicylic acid (5-ASA) preparations, corticosteroids, immunomodulators, biologics, and small molecules, as well as cell apheresis therapy (CAP) and surgical interventions. These therapies are chosen according to disease severity and activity. Furthermore, in CD, enteral nutrition therapy using elemental nutritional supplements has been shown to be effective in inducing remission and improving intestinal lesions. In contrast, in many cases, UC tends to be relatively milder than CD, and there is limited evidence regarding the effectiveness of dietary therapy during remission phases [1].

In Japan, the prevalence of UC has increased steadily over the past decades. A nationwide survey in 2023 estimated approximately 316,900 patients with UC, with a prevalence of 254.8 cases per 100,000 population, representing a 1.4‑fold increase compared with the 2015 survey [2]. UC and CD are designated as intractable and rare diseases under Japan’s health system. However, following the 2015 revision of the medical expense subsidy system, although the Japanese government did not provide an explicit explanation for excluding mild cases of UC from the intractable disease subsidy system, two factors are considered relevant. First, the number of patients diagnosed with UC has increased steadily, raising concerns over the sustainability of public financial support. Second, with the advancement and widespread use of anti-inflammatory agents such as 5-ASA, remission induction and maintenance have become more manageable in mild cases compared to the past. These developments may have led policymakers to deprioritize financial assistance for patients with less severe disease, despite UC remaining an intractable condition. Patients with mild UC were excluded from financial support, underscoring the growing importance of self-care in this population. Nurses play a role in supporting these patients in acquiring, implementing, and maintaining self-care abilities. Nursing interventions help reduce emergency visits and hospitalizations due to acute exacerbations, contributing to the maintenance and improvement of patients' quality of life.

Self-care refers to actions by which individuals actively attend to their health, including disease management and recovery behaviors, while flexibly adapting to their own life circumstances. It also includes health-promoting and disease-preventing activities such as self-monitoring and self-management [3]. Daily monitoring of diet and physical condition enables patients to objectively recognize symptoms and changes, thereby facilitating early intervention. Moreover, self-care enhances patients’ sense of self-efficacy and encourages active engagement in their own treatment [4].

A 2016 literature review of nursing research on patients with IBD in Japan revealed that most studies focusing on UC were case studies, limited to understanding individual patients’ specific circumstances and their commonalities [5]. However, the current state of nursing care and patient education aimed at supporting self-care among patients with UC in Japan remains unclear. Therefore, this study aims to assess current nursing care practices and patient education provided by nurses to patients with UC in Japan using a scoping review. Much of the existing research has been limited to case reports and descriptive studies, with insufficient intervention studies and effectiveness evaluations. Therefore, we determined that a scoping review, which organizes key concepts and research methods within the field to identify knowledge gaps and future research directions, is more appropriate than a systematic review. The findings of this study are expected to serve as foundational material for supporting self-care in patients with UC and contribute to the development of educational programs by nurses.

## Review

Methods

Study Design

A scoping review was done in accordance with the methodological framework proposed by Arksey and O’Malley and the Preferred Reporting Items for Systematic Reviews and Meta-Analyses extension for Scoping Reviews (PRISMA-ScR) guidelines [6,7].

Extraction of Target Papers

Identification of research questions: Three researchers discussed and defined the research questions to explore the current state of nursing care and patient education provided by nurses to patients with UC. The research parameters were set according to the PCC framework as follows: the Population was patients with UC; the Concept was nursing care and patient education provided by nurses; and the Context was Japan, including both hospital and community settings, with no restrictions on disease severity.

Identification of key studies: For the literature search, Japan Medical Abstracts Society (Ichu-Shi Web) (https://login.jamas.or.jp/) and CiNii (https://cir.nii.ac.jp/) were used. Ichushi-Web is Japan’s most comprehensive medical database, ensuring broad domestic coverage. The search was conducted on June 19, 2025, and covered studies published between 2015 and 2025.

To clarify the search strategy, we selected the keywords “nursing”, “self-management”, and “patient education” to explore how nurses, who play a central role in patient education, support patients with UC in acquiring self-management behaviors. We also included “diet therapy” as a keyword based on previous reports that many patients with UC impose dietary restrictions during remission despite the lack of strong evidence. The search was limited to Japanese-language databases because the review aimed to capture nursing interventions and education practices reported in Japan, which are predominantly published in Japanese.

In Ichushi-Web, the search was limited to the nursing category and restricted to original research articles. The search formula combined thesaurus terms (TH) and free terms (AL) using the logical operator AND. CiNii was searched using the same keywords, excluding materials of limited academic value such as explanatory articles, special issues, and conference proceedings.

Eligibility Criteria and Study Selection

Studies targeting patients with UC or their families that described nursing care or patient education provided by nurses and clearly outlined the content of nursing interventions were included. Studies involving other healthcare professionals were included only if they met the aforementioned criteria. Literature review articles were excluded, and study quality was not assessed, as the aim of this scoping review was to map existing evidence rather than evaluate intervention effectiveness. To better capture the clinical context, literature published in regional academic journals was also included.

During the primary screening, three researchers independently and simultaneously searched for and reviewed potentially relevant studies, selecting those whose titles and abstracts met the eligibility criteria. In the secondary screening, full texts of the selected articles were retrieved, and two researchers independently evaluated whether each study fulfilled the inclusion criteria.

In cases of disagreement, the researchers discussed the discrepancies and reached a consensus on whether to include or exclude the study.

Data Extraction, Synthesis, and Analysis

Data extraction: The selected studies were organized by author, year of publication, journal, research design, research objective, characteristics of study participants, treatment details, evaluation measures and analytical methods, intervention details, and main findings.

Data collection, summarization, and reporting: Each study identified through the search was carefully reviewed, and content related to nursing care and patient education provided by nurses to patients with UC and their families was extracted.

For data extraction and coding, the extracted information was synthesized into a single representative sentence per article, which was then used as a coding unit. These units were categorized based on thematic similarity and difference, and grouped into overarching categories and themes. We ensured that the coding preserved the original meaning while providing concise and comparable expressions across studies. Two researchers independently extracted and categorized the nursing care and patient education content, grouping similar items into categories and themes. This analysis was conducted in accordance with the PRISMA-ScR guidelines [8].

Ethical Considerations

The handling of source materials was carried out with due care to avoid copyright infringement and to ensure fidelity to the original texts. An ethical review was not required for this study.

Results

Overview of the Selected Literature

**Figure 1:**
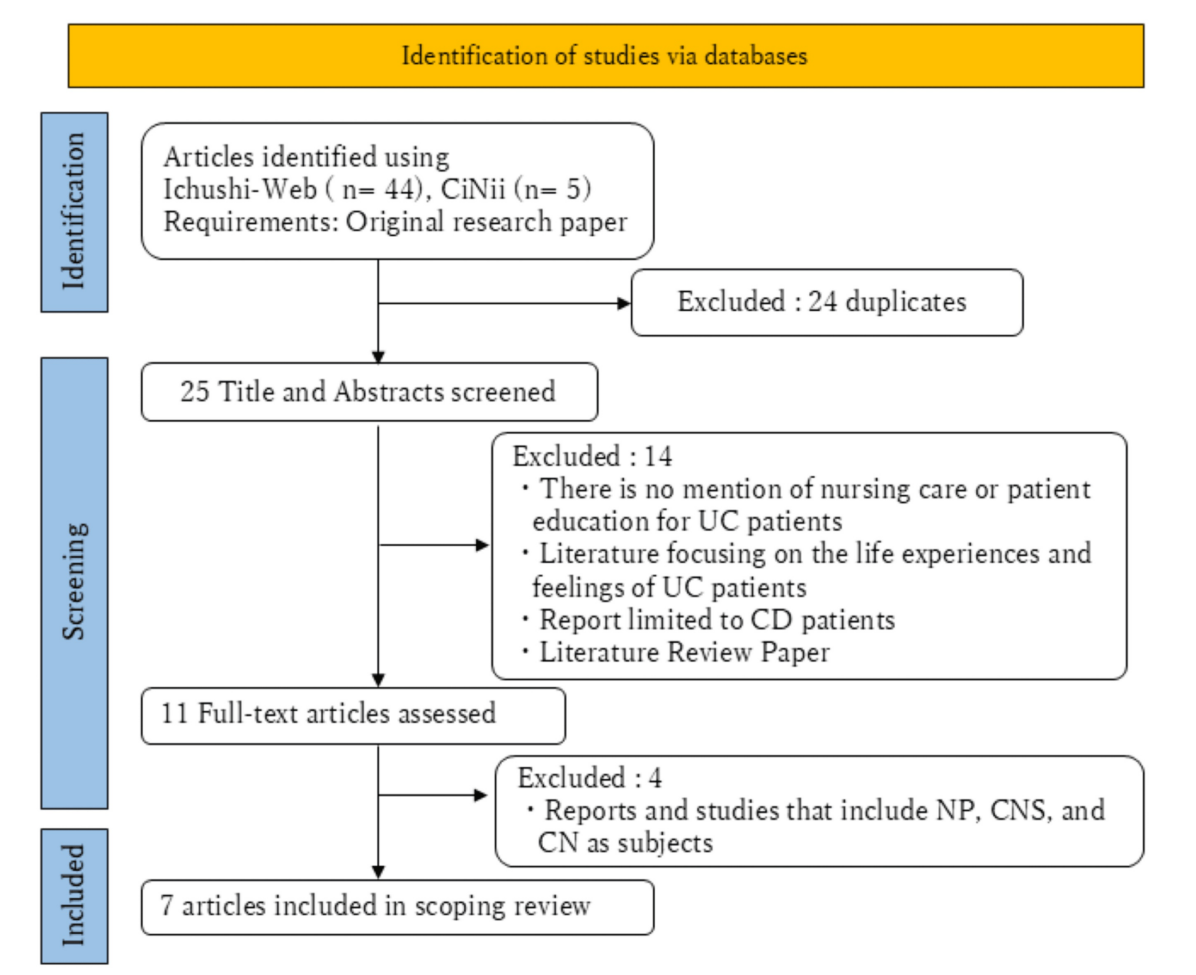
Preferred Reporting Items for Systematic Reviews and Meta-Analyses extension for Scoping Reviews (PRISMA-ScR) flowchart UC, ulcerative colitis; CD, Crohn’s disease; NP, nurse practitioner; CNS, certified nurse specialist; CN, certified nurse

The literature search initially identified 49 papers, of which 24 duplicates were excluded. During the primary screening, 14 papers were excluded based on the eligibility criteria, leaving 11 papers for full-text review. After the secondary screening, four additional papers were excluded, resulting in a total of seven papers included in the final analysis (Figure 1).

The publication years of the selected studies were as follows: 2016 (two papers), and 2017, 2019, 2020, 2022, and 2023 (one paper each). The journals of publication included three academic society journals, two conference proceedings, and two medical journals (Table 1). Regarding study design, six were case reports, and one was a survey study.

**Table 1:** Studies included in the review

Number	Title	Author (Year)	Journal	Study Design
1	Supporting Patients in Their 20s Who Undergo Permanent Stoma Surgery Toward Acceptance	Kohira et al. (2016) [9]	Nagano Nursing Research Association Journal	Case report
2	Nursing Care for a Patient with Ulcerative Colitis Complicated by Pyoderma Gangrenosum: Reflecting on the Process of Accepting Steroid Treatment	Noguchi et al. (2016) [10]	Annals of Nursing Research, Shinshu University Hospital	Case report
3	Psychological Follow-Up for Patients Who Underwent Surgery Following the Onset of Ulcerative Colitis	Akiyama (2017) [11]	Nagano Nursing Research Association Journal	Case report
4	Informed Consent Preparation for Pediatric Patients With Ulcerative Colitis	Suekichi (2019) [12]	Okinawa Prefecture Nursing Research Society Proceedings	Case report
5	Survey on Actual Usage of Syringe and Pen Formulations of Adalimumab Subcutaneous Injection, an Anti-TNF-α Antibody Agent, in IBD Patients	Sakagami et al. (2020) [13]	Progress in Medicine	Survey
6	Nursing Interventions to Prevent Crises in Patients With Ulcerative Colitis Who Are Being Considered for Surgical Treatment: Consideration Using Aguilara-Mezwick's Crisis Theory	Hiyamuta and Nakazato (2022) [14]	Fukuoka Red Cross Nursing Research Society Proceedings	Case report
7	The Reality of Support for Rare Disease Care. Part 2: Multidisciplinary Team Practice for a Patient with Refractory Ulcerative Colitis and a History of Parkinson's Disease	Ebisawa (2023) [15]	Home Health Care for the People with Intractable Disease	Case report

All case reports described nursing care and patient education for hospitalized patients with UC (Table 2); one case involved the active phase, while five involved recurrent relapses. Four reports focused on colostomy or stoma management. The age of patients in these reports ranged from their teens to their 60s. The survey study examined the actual use and challenges of self-injection of biologic agents among patients over teens, as well as their evaluation of nursing guidance. 

**Table 2:** Overview of the literature M, male; F, female; NA, not applicable; UC, ulcerative colitis; ADL, activities of daily living; WOCN, Wound, Ostomy and Continence Nurse; FPS, Faces Pain Scale; GCAP, granulocyte and monocyte adsorption apheresis; NST, nutrition support team; PT, physical therapist

Number	Age/Sex	Stage	Treatment for UC	Nursing Intervention	Result
1	20s/M	Relapse Active	Oral medication: details unknown. Surgical treatment: permanent stoma creation, anal closure	Explanation of ostomy care supplies and methods by the WOCN, stoma care (device replacement, managing leakage of waste), strategies for stoma self-management and education with personalized pamphlets, closed wound dressing change, innovative approaches and education for self-management of wound care, pain management, consultations and coordination with palliative care teams and plastic surgery, establishing a system for ongoing support after discharge and introducing patient support groups, emotional expression, active listening, and other forms of psychological support	Discharged five weeks after surgery, acquisition of stoma care skills before discharge, acquisition of the gauze dressing change technique for anal closure wound, improved psychological stability and increased positive expressions
2	40s/M	Relapse Active	Medical treatment: steroids, immunosuppressants, traditional Kampo medicine	Listen to the patient’s feelings about steroid therapy, explore the process leading to the refusal of steroid treatment and the wife’s perspective, request a pharmacist to provide medication counseling when introducing immunosuppressive agents, pain management including medication management and pain relief, assist with hygiene care (including elimination) according to the degree of pain, provide wound care for leg ulcers, provide guidance on self-management of medication and wound care for the lower limbs, coordinate patient education provided by physicians and pharmacists	Improvement of lower limb pain, recovery of ADL, including transfer to and ambulation to the portable toilet, acceptance of treatment with steroids and immunosuppressive agents, selection of combined therapy with steroids and Kampo medicine after discharge
3	60s/F	Active	Medical treatment: immunosuppressants, leukocyte depletion therapy, pain management, opioid medication. Surgical treatment: laparoscopic total colectomy with temporary colostomy creation	Conduct stoma orientation, including explanations about stoma care and social reintegration	Underwent surgery successfully, acquisition of stoma care skills in cooperation with her husband, softer facial expressions and increased smiling
4	Teens/NA	Relapse Active	Medical treatment: central venous nutrition, fecal microbiota transplantation, immunosuppressants, leukocyte depletion therapy, pain management. Surgical treatment: total colectomy with temporary colostomy	Monitor pain using FPS for pain management, pain management through multidisciplinary collaboration, explanation using pamphlets, coordination for the observation of stoma patients, providing supplementary explanations using easy-to-understand language, anatomical illustrations, and associative cues (e.g., color or smell of medication) after the physician’s explanation, speaking on behalf of the child to convey their feelings and thoughts about surgery	Pain relief through modification of pain assessment and analgesic regimen, expression of emotions and opinions after observing another patient, reduction of anxiety regarding postoperative condition, decision to undergo surgery facilitated by acceptance of the patient’s thoughts and preferences in treatment planning
5	Fifteen patients ranging in age from their teens to their 60s/M=8, F=7	NA	NA	Have the patient watch instructional videos and materials in advance regarding the procedure for self-injection, provide detailed instructions, including hands-on guidance and points of caution	Significant increase in the proportion of patients who rated the educational program as “very helpful” after receiving nurses’ explanations following video and material viewing (from 37.4% to 54.9%)
6	30s/M	Relapse Active	Medical treatment: change and adjustment of medications, introduction of Remicade® and GCAP	Social Support Role: listen to the patient’s thoughts and feelings after obtaining surgical informed consent, understand the patient’s social background, beliefs, and values that influence treatment decisions. Multidisciplinary Coordination Role: share information with the attending physician and the surgical team regarding postoperative life and the patient’s desire for detailed information about diet and treatment, coordinate postoperative dietary guidance by a dietitian, request intervention from a WOCN, prepare stoma-related pamphlets and stoma samples	Cancellation of surgery due to clinical improvement with Remicade and GCAP therapy
7	60s/F	Relapse Active	NA	Assist with mobility (such as sitting up) considering fluctuations due to Parkinson’s disease and movement difficulties, share information with PTs, pharmacists, and the attending physician to coordinate a neurology consultation and adjust anti-Parkinsonian medication, pain management, collaborate with the NST, provide self-care education (medication adherence, physical condition management, and topical medication management)	Resumption of oral intake, self-management with an assistive device for enema foam application, transfer for rehabilitation for home discharge

Nursing Care and Patient Education Content for Patients With UC

Codes representing nursing content extracted from the literature were grouped according to similarity. Eight categories and four overarching themes of nursing care and patient education provided by nurses were identified: “Stoma Management and Care”, “Guidance on Self-Management of Medication and Wound Care”, “Collaboration and Coordination with Other Professionals”, “Pain Management”, “Wound and Ulcer Care”, “Assistance with Activities of Daily Living”, “Patient Understanding and Advocacy Role”, and “Psychological Care” (Table 3).

**Table 3:** Nursing interventions and patient education for patients with UC UC, ulcerative colitis; WOCN, Wound, Ostomy, and Continence Nurse; NST, nutrition support team; PD, Parkinson's disease

Theme	Category	Code	Number
Self-Management Education and Technical Guidance	Stoma Management and Care	Request for intervention by WOCN	6
		Guidance on selecting and using ostomy care supplies by a WOCN	
		Preparation of pamphlets and stoma samples related to colostomies	1
		Stoma care (device replacement, managing leakage)	
		Self-management techniques and education (creating personalized pamphlets)	
		Stoma orientation (explanation of knowledge about colostomies and returning to society)	3
		Arranging visits for patients undergoing stoma creation	4
		Explanations in the pamphlet	
	Guidance on Self-Management of Medication and Wound Care	Self-care guidance included oral medication, management of physical condition, and management of topical medication	7
		Strategies and support for self-administering wound care	1
		Instruction on self-management for lower limb care and medication	2
		Viewing videos and materials explaining the entire process of self-injection administration	5
		Technical guidance and explanation of points to note regarding biological products	
Multidisciplinary Collaboration	Collaboration and Coordination with Other Professions	Sharing information about the patient's wishes with the primary physician and the surgeon in charge	6
		Adjustment of postoperative dietary guidance by a nutritionist	
		Share information with physical therapists, pharmacists, and attending physicians; coordinate consultations with other departments and medication adjustments	7
		Collaboration with the NST	
		Consultation with the palliative care team or plastic surgery department	1
		Establishing a system for ongoing support after discharge and introducing patient support groups	
		Request for pharmacist medication counseling when initiating immunosuppressants	2
		Coordinating guidance from physicians and pharmacists	
		Pain management (shared with multidisciplinary teams)	4
Direct Care	Pain Management	Pain management	1,7
		Pain management (medication management, pain relief)	2
		Pain management (change in assessment method)	4
	Wound and Ulcer Care	Closed wound dressing change	1
		Lower limb ulcer care	2
	Assistance with ADL	Assistance with sitting up due to PD-related fluctuations throughout the day or difficulty moving the body	7
		Assistance with toileting (elimination) tailored to the level of pain	2
Patient Understanding and Emotional Support	Patient Understanding and Advocacy Role	Listening to the patient's thoughts after the preoperative consultation	6
		Understanding the patient's social background, beliefs, and values	
		Listen attentively to the individual's thoughts regarding adrenal corticosteroids	2
		Listen to the circumstances leading to the refusal of steroid treatment and the wife's feelings	
		Listening to the child's feelings about surgery and advocating for their thoughts	4
	Emotional Care	Emotional support such as expressing feelings and active listening	7
		After the physician's explanation, a follow-up discussion to ensure the patient's understanding	4

Self-Management Education and Practical Instruction: This theme comprised two categories: “Stoma Management and Care” and “Guidance on Self-Management of Medication and Wound Care”.

Multidisciplinary Collaboration: This theme was identified as a category under “Collaboration and Coordination with Other Professions”. The collaborating professionals included attending physicians, surgeons, pharmacists, dietitians, and physical therapists.

Direct Care: This theme comprised three categories: “Pain Management”, “Wound and Ulcer Care”, and “Assistance with ADL”.

Patient Understanding and Emotional Support: This theme yielded two categories: “Patient Understanding and Advocacy Role” and “Emotional Care”.

Discussion

Although Japanese dietary habits are often regarded as healthier than those of Western countries, recent trends show increasing Westernization of dietary patterns. Some studies suggest that this shift may be contributing to the rising incidence of ulcerative colitis, particularly among middle-aged and older adults. However, there remains no established evidence regarding the effectiveness of dietary therapy during remission in ulcerative colitis, regardless of cultural context. Therefore, while cultural dietary differences may influence disease perception or patient behaviors, the current review focused on nursing interventions and patient education rather than cultural dietary factors.

Actual State of Nursing Care and Patient Education for UC Patients

In the present review, a total of seven studies were identified from 2015 to 2025, of which six were case reports published in regional Japanese journals. This predominance of case-based publications is consistent with a previous review of UC nursing research in Japan by Kawauchi et al. [5], which also reported that more than 90% of identified studies were case reports. Furthermore, Kawauchi et al. reported that about 40% of the studies addressed stoma care, and similarly, stoma care was the most frequently reported topic in the present review [5].

None of the included studies explicitly stated the clinical severity of UC. However, based on treatment context and target population, all studies appeared to involve patients with moderate to severe disease. Interventions were not stratified by severity, highlighting a gap in the literature.

Although this review focused on the Japanese-language literature, the limited number of systematic reviews on nursing interventions and patient education for patients with UC suggests that the lack of scientific reporting is not unique to Japan. However, in Japan, such practices are often embedded within physician-led treatment and may not be formally documented in the academic literature.

Education on Self-Management, Including Stoma Care and Medication Management

Whether temporary or permanent, stoma creation causes significant psychological shock and often places patients in a state of crisis. After surgery, patients must acquire and maintain appropriate management techniques on their own. Therefore, nursing care that alleviates psychological distress, facilitates acceptance of the stoma, and provides practical support for stoma management has been identified as an area of high clinical importance. In addition, two studies reported that nurses sought the involvement of WOCNs (Wound, Ostomy, and Continence Nurses) and collaborated with them. WOCNs are nurses certified by the Japan Nursing Association who possess advanced expertise in stoma care and deliver high-quality nursing interventions [16]. The selection and implementation of appropriate stoma products and management strategies under WOCN guidance were considered effective in promoting patient recovery and the acquisition of self-management skills.

Beyond stoma care, several studies described nursing interventions that aimed to improve patients’ treatment-related self-management skills, including wound care, oral medication administration, and self-injection of biologic agents. Medication management, encompassing both oral drugs and biologics, is a critical aspect of daily treatment that requires consistent adherence to maintain therapeutic efficacy. Among patients with UC with a disease duration exceeding one year, the non-adherence rate for 5-ASA preparations was reported to be 59.4%, with poorer adherence observed in younger patients; the risk of relapse was 2.3 times higher compared to the adherent group [17,18]. Thus, providing patients with individualized education and strategies tailored to their lifestyle, from hospitalization through discharge, is considered a key component of effective nursing care.

Patient Understanding and Psychological Support

Surgery and stoma creation cause patients to experience an internal conflict between the fear of surgery and changes in body image that shake their sense of self, and the recognition that treatment is necessary to prevent further deterioration or progression of the disease [19]. Similarly, corticosteroids, though highly effective in controlling inflammation, are also associated with numerous adverse effects; as a result, patients often develop ambivalent or negative feelings toward their use. In response, nurses implemented practices that acknowledged and validated patients’ complex emotions and provided care with an empathetic attitude. To ensure that their empathy was not merely superficial, nurses listened carefully to patients’ backgrounds, feelings, and inner conflicts. Through continuous dialogue, they sought to understand patients’ individual values and beliefs, demonstrating a genuine commitment to engaging with them.

Rogers (1995) described the empathetic attitude as “to sense the client’s private world as if it were your own, but without ever losing the ‘as if’ quality - this is empathy, and this seems essential to therapy”[20]. This stance defines the nurse’s way of being in a helping relationship. Across the case reports, nurses’ involvement included both empathetic listening and psychological support aimed at alleviating patients’ anxiety and fear. Such approaches represented essential nursing competencies that supported patients’ self-determination and collaborative engagement in their treatment with healthcare providers.

Multidisciplinary Support and Direct Care

To address the complex and diverse challenges faced by patients, multidisciplinary, team-based care involving physicians, nurses, dietitians, pharmacists, and other healthcare professionals has been increasingly promoted in recent years. Among the studies reviewed, five reported consultations with professionals other than physicians, such as pharmacists, dietitians, and physical therapists, as well as with multidisciplinary palliative care teams and nutrition support teams, with nurses playing a central coordinating role.

The support provided included sharing information regarding patients’ thoughts and wishes, adjusting medications and providing medication management guidance, offering dietary counseling, and implementing pain management interventions. Pain management, in particular, represents a core aspect of direct nursing care and requires specialized knowledge and skills, including medication adjustment.

Challenges in Nursing Care for UC Patients

UC is a chronic condition that typically develops at a young age, has no definitive cure, and requires lifelong treatment and disease management. Therefore, chronic disease self-management programs may also be effective for UC. Such programs include components such as exercise, symptom management, nutrition, fatigue and sleep management, medication adherence, stress management, resource utilization, and problem-solving and decision-making. For various chronic diseases, self-management programs have been shown to improve health outcomes by promoting health behaviors, increasing social participation, and reducing hospital visits [21]. Additionally, studies have reported that self-management in IBD improves both intestinal and systemic symptoms, potentially leading to better disease control and outcomes [22]. Biweekly group sessions focusing on self-management have also been found to enhance self-efficacy and quality of life in patients with UC [4]. The present review revealed that nursing care for patients with UC in Japan primarily focuses on direct care, such as stoma management, and psychological support. Educational interventions related to self-care were limited to stoma management, wound care, medication management, and self-injection techniques. Previous studies examining lifestyle adjustments among UC patients identified categories such as awareness of the impact of abdominal symptoms on daily life, actions taken based on experience to prevent symptom exacerbation, and preparation for urgent bowel movements as common coping behaviors in everyday life [23]. However, the findings of this review did not include such lifestyle adjustment strategies. Therefore, it is necessary to provide UC patients with appropriate information and education regarding daily life self-care, including both its content and methods. Such support should involve practical and collaborative problem-solving with patients, enabling them to manage their physical condition and stress, and to take effective coping actions when needed.

Although dietary therapy is generally not required for patients with mild or remission-phase UC, appropriate patient education on dietary management remains essential. Indeed, more than half of patients with UC, even during remission, engage in self-care behaviors such as avoiding specific foods based on experiences of diet-induced abdominal pain or diarrhea [24]. For CD patients, component nutrition therapy has been shown to be effective in preventing reoperation, and adherence to an anti-inflammatory diet during remission has been associated with a higher rate of maintaining clinical remission [25,26]. Therefore, dietary adjustments are recognized as an important aspect of self-care in IBD, although their role and implementation differ by disease. In contrast, in patients with UC, dietary modifications need to be made according to disease activity. Accordingly, nurses should carefully explore each patient’s dietary experiences, regularly assess their nutritional status, and provide individualized and comprehensive self-care support that also addresses the importance of maintaining a healthy diet.

Strengths and Limitations

The strength of this study lies in its assessment of the current characteristics of nursing practice for patients with ulcerative colitis in Japan. Using a scoping review of the Japanese-language literature, we identified that nursing care has predominantly focused on stoma care and psychosocial support for patients with severe disease. This study also highlights important gaps in the literature, including the lack of research examining differences in nursing practices by disease severity and the limited evaluation of the effectiveness of nursing interventions.

This study has several limitations as well. The review was restricted to the Japanese-language literature and used only two databases (Ichushi-Web and CiNii), so relevant publications may not have been comprehensively captured. Although the included studies consisted mainly of case reports, this scoping review was not restricted to case reports by design. We conducted a comprehensive search excluding conference abstracts and narrative commentaries; however, no other empirical studies addressing nursing interventions or patient education for patients with UC in Japan were identified. While case reports have limited generalizability, they provide detailed descriptions of clinical nursing practices and represent the best available evidence in a field where systematic or quantitative studies remain scarce. Moreover, all selected studies were descriptive reports published in regional academic journals, lacking intervention studies or evaluations of nursing effectiveness. In addition, data extraction and classification by two researchers inevitably involved subjective judgment. Given these limitations, future studies should systematically investigate and evaluate the current state of self-care practices among UC patients, as well as the content and effectiveness of nurse-led self-care education. Such research will contribute to the development of a comprehensive, evidence-based nursing care model for UC.

## Conclusions

Nursing care and patient education for patients with UC encompassed self-management education and practical guidance, multidisciplinary collaboration, direct care, and patient understanding and psychological support. These nursing practices may have clinical utility in improving patients’ quality of life and treatment adherence by alleviating acute conditions, facilitating stoma acceptance, and ensuring appropriate wound care and pain management. However, most existing studies are descriptive in nature, and few have empirically examined the effectiveness of nursing interventions. Future research should systematically investigate the current status and outcomes of nurse-provided self-care education. Such studies could contribute to the development of nurse-led educational programs and nursing approaches tailored to patients’ life stages and disease phases, ultimately establishing a comprehensive care model for patients with UC.
